# Dexmedetomidine Improves Anxiety-like Behaviors in Sleep-Deprived Mice by Inhibiting the p38/MSK1/NFκB Pathway and Reducing Inflammation and Oxidative Stress

**DOI:** 10.3390/brainsci13071058

**Published:** 2023-07-11

**Authors:** Jiangjing Li, Heming Zhang, Bin Deng, Xin Wang, Peng Liang, Shenglong Xu, Ziwei Jing, Zhibin Xiao, Li Sun, Changjun Gao, Jin Wang, Xude Sun

**Affiliations:** 1 Department of Anesthesiology, The Second Affiliated Hospital of Air Force Medical University, Xi’an 710038, China; lulu2790@163.com (J.L.); gaocj74@163.com (C.G.); sunxudes@163.com (X.S.); 2 Department of Anesthesiology & Center for Brain Science, The First Affiliated Hospital of Xi’an Jiaotong University, Xi’an 710065, China; 3 Department of Otolaryngology Head and Neck Surgery, Shaanxi Provincial People’s Hospital, Xi’an 710068, China; 4 Department of Rehabilitative Physioltherapy, The Second Affiliated Hospital of Air Force Medical University, Xi’an 710038, China; 5 Department of Radiation Medical Protection, Ministry of Education Key Lab of Hazard Assessment and Control in Special Operational Environment, School of Military Preventive Medicine, The Fourth Military Medical University, Xi’an 710068, China; 6 Department of Anesthesiology, The 986th Air Force Hospital, Xijing Hospital, The Fourth Military Medical University, Xi’an 710032, China

**Keywords:** dexmedetomidine, sleep deprivation, neuroinflammation, p38 MAPK, oxidative stress

## Abstract

(1) Background: Sleep deprivation (SD) triggers a range of neuroinflammatory responses. Dexmedetomidine can improve sleep deprivation-induced anxiety by reducing neuroinflammatory response but the mechanism is unclear; (2) Methods: The sleep deprivation model was established by using an interference rod device. An open field test and an elevated plus maze test were used to detect the emotional behavior of mice. Mouse cortical tissues were subjected to RNA sequence (RNA-seq) analysis. Western blotting and immunofluorescence were used to detect the expression of p38/p-p38, MSK1/p-MSK1, and NFκBp65/p- NFκBp65. Inflammatory cytokines were detected using enzyme-linked immunosorbent assay (ELISA); (3) Results: SD triggered anxiety-like behaviors in mice and was closely associated with inflammatory responses and the MAPK pathway (as demonstrated by transcriptome analysis). SD led to increased expression levels of p-p38, p-MSK1, and p-NFκB. P38 inhibitor SB203580 was used to confirm the important role of the p38/MSK1/NFκB pathway in SD-induced neuroinflammation. Dexmedetomidine (Dex) effectively improves emotional behavior in sleep-deprived mice by attenuating SD-induced inflammatory responses and oxidative stress in the cerebral cortex, mainly by inhibiting the activation of the p38/MSK1/NFκB pathway; (4) Conclusions: Dex inhibits the activation of the p38/MSK1/NFκB pathway, thus attenuating SD-induced inflammatory responses and oxidative stress in the cerebral cortex of mice.

## 1. Introduction

Sleep is critical for health and normal brain function. Sleep deprivation (SD)—defined as inadequate sleep below baseline requirements—is known to affect overall health and wellness. SD reduces immune function, cognitive memory, and learning ability and disrupts emotional health, thereby affecting the daily life activities of individuals [1,2,3,4]. SD impairs the functioning of the sympathetic nerve system, leading to metabolic dysregulation [5]. In addition, SD triggers a range of neuroinflammatory responses that modulate immune function by increasing the release of pro-inflammatory cytokines such as interleukin-1β (IL-1β), interleukin-6 (IL-6), tumor necrosis factor (TNF-α), and C-reactive protein (CRP) [6,7]. Growing evidence suggests that SD-induced anxiety behaviors should be closely related to the activation of astrocytes and microglia in the central nervous system (CNS), leading to increased levels of proinflammatory markers and nerve damage [6,8,9,10]. Although neuroinflammatory responses and oxidative stress are both key factors related to the adverse effects of SD, little is known about the regulatory mechanisms that mitigate these factors [11].

Dexmedetomidine (Dex) is a potent and selective agonist of α2-adrenergic receptors that has seen widespread clinical use since its approval by the US Food and Drug Administration in 1999. Dex exerts protective effects on the nervous system, maintains anesthetic activity, and attenuates immune suppression without causing respiratory depression [12]. Dex has been shown to have neuroprotective effects both in vivo and in vitro [13,14,15] and these effects are increasingly considered to have clinical implications. Because Dex sedation is closer to the characteristics of natural sleep [16], Dex is often used to improve the sleep quality of perioperative patients and critically ill patients [17,18,19,20,21]. Previous studies have shown that dexmedetomidine can improve the emotional behavior [22] and cognitive dysfunction [23] caused by sleep deprivation but the mechanism is not clear. Recent studies have shown that mice deprived of acute rapid eye movement (REM) sleep for 3 days show increased expression of IL-17A and IL-17f and activation of the p38 MAPK pathway in the hippocampus [24]. In addition, the p38 signaling pathway is involved in SD-induced activation of the NLRP3/pyroptosis axis [25]. Therefore, we hypothesized that Dex might alleviate the anxiety behaviors of sleep-deprived mice by reducing the inflammatory response through the p38 MAPK pathway. In this study, we investigated the effects of Dex on the inhibition of inflammatory response pathways in sleep-deprived mice. We evaluated Dex-induced improvements in emotional behavior in sleep-deprived mice. Our results provide support for the use of Dex in the treatment of sleep disorder-related diseases.

## 2. Materials and Methods

### 2.1. Induction of SD

**Figure 1:**
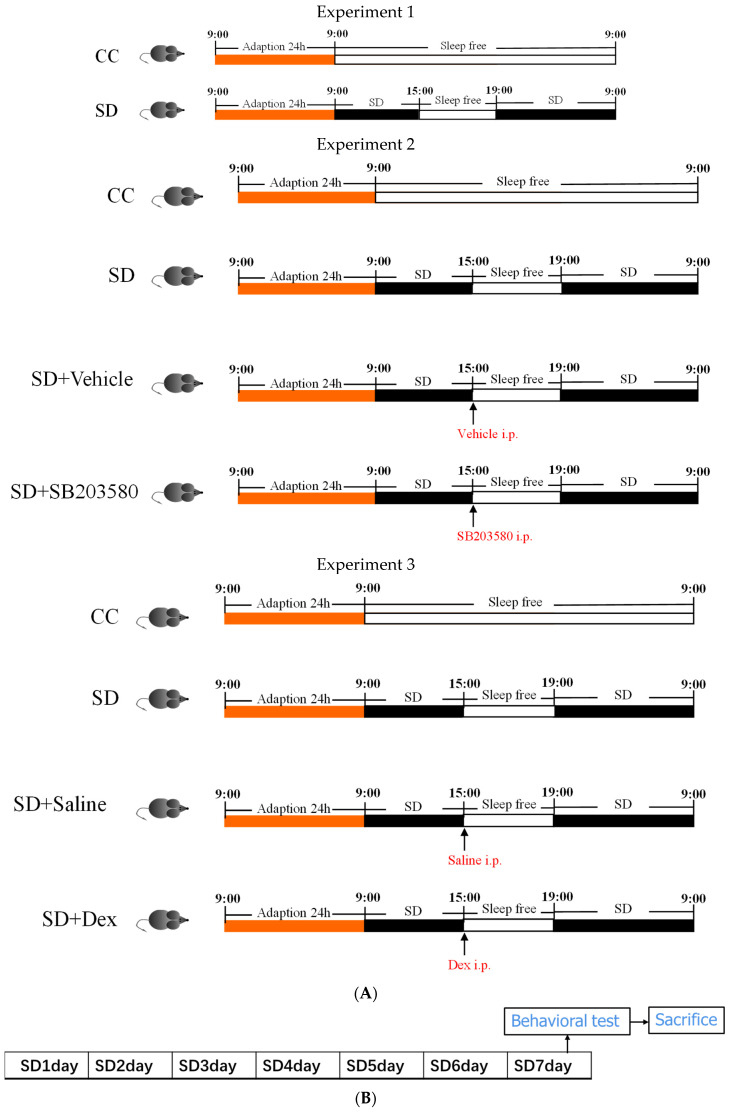
Establishment of the sleep deprivation models. (**A**) Single-day sleep deprivation model with 4 h of sleep every 24 h (during the last 4 h of the light phase) in Experiment 1, Experiment 2, and Experiment 3. (**B**) Seven-day sleep deprivation model.

The animal model of SD was established (ZL-013, Anhui Yaokun Biotechnology Co., Ltd., Hefei, China) [6]. Male C57BL/6 J mice were placed in a transparent Plexiglas cylinder (400 mm × 390 mm) and allowed to move, feed, and drink water freely. The cylinder contained a horizontal bar at the bottom that rotated in a random direction at a speed of 5 rpm. The duration of SD was 7 days, with only 4 h of sleep per 24 h (during the last 4 h of the light period). Specifically, the sleep disruption bar was rotated for 20 h per day, and the rotation was halted during 15:00–19:00 each day to allow the mice to sleep. This was continued for 7 days. The procedures of Experiment 1, Experiment 2, and Experiment 3 are shown in Figure 1A. Experimental protocols were approved by the Medical Experimental Animal Administrative Committee of Air Force Medical University (No. IACUC-20210963) and strictly followed the Guidelines from the National Institute of Health (U.S.) regarding the care and use of animals for experimental procedures. Every effort was made to minimize the number of animals for experiments and any pain or discomfort they experienced.

### 2.2. Experimental Animals and Pharmacological Treatments

Male C57BL/6 J mice aged 8–10 weeks were used in the experiments. All animals were purchased from the Animal Research Center of the Air Force Medical University. The mice were reared under controlled conditions (ambient temperature, 23 °C; 12-h photoperiod with illumination during 7:00–19:00) and were provided with water and food ad libitum. In Experiment 1, mice were randomly divided into two groups: the control cage (CC) group and the sleep deprivation (SD) group. In Experiment 2, mice were randomly divided into four groups: the control cage (CC) group and the sleep deprivation (SD) group, the sleep deprivation and SB203580 (SD+SB203580; daily intraperitoneal injection of 0.5 mg/kg SB203580 [26] for 6 days) group, and the sleep deprivation and vehicle (SD + vehicle; intraperitoneal injection of vehicle equal in volume to SB203580) group. SB203580 (0.5 mg/kg) was purchased from MedChemExpress Co., Ltd. (Shanghai, China). The vehicle was 0.1% DMSO. In Experiment 3, the animals were divided into four groups: the control cage (CC) group, the sleep deprivation (SD) group, the sleep deprivation and dexmedetomidine (SD + Dex; daily intraperitoneal injection of 100 μg/kg Dex for 6 days) group, and the sleep deprivation and saline (SD + saline; intraperitoneal injection of saline equal in volume to Dex) group [22,23]. Dex was purchased from Yangtze River Pharmaceutical (Group) Co., Ltd. (Taizhou, China). Mice were anesthetized with an O_2_ −2% isoflurane mask before specimen collection.

### 2.3. Open-Field Experiment

In a quiet environment, the mice were introduced into a 50 cm × 50 cm open field from a fixed position and allowed to move freely. Their activities and movements were recorded for 10 min and the data were extracted for analysis. After each test, the field was cleaned of feces and urine stains and wiped with alcohol before another mouse was introduced. The central area was set as the target area, we mainly analyzed the duration of mice in the target area. The average speed of movement of the mice was used to judge whether the model and treatment affected the movement ability of the mice. The behavioral experimental data were recorded using Supermaze software, which was provided by Shanghai XinRuan Information Technology Co., Ltd. (Shanghai, China). 

### 2.4. Elevated plus Maze Experiments

In a quiet environment, the mice were gently placed in the center of an elevated plus maze (arm width, 5 cm; arm length, 35 cm; closed arm height, 15 cm; maze height, approximately 40–55 cm above the ground). The mice were oriented to face the open arm and were allowed to roam, and their activities were recorded for 5 min. After each test, the maze was cleaned of feces, urine stains, and other debris and wiped with alcohol before another mouse was introduced. The open arm was set as the target area, we mainly analyzed the duration of mice in the target area. The average speed of movement of the mice was used to judge whether the model and treatment affected the movement ability of the mice. The behavioral experimental data were recorded using Supermaze software, which was provided by Shanghai XinRuan Information Technology Co., LTD (Shanghai, China).

### 2.5. mRNA Sequencing

RNA sequencing was performed on mice cortical tissue. Total RNA was isolated from each sample using the standard TRIzol protocol (Invitrogen, Carlsbad, CA, USA). RNA quality was examined using gel electrophoresis and with a Nanodrop spectrophotometer (Thermo, Waltham, MA, USA). Strand-specific libraries were constructed using the TruSeq RNA sample preparation kit (Illumina, San Diego, CA, USA). The libraries were sequenced by Genergy Biotechnology Co., Ltd. (Shanghai, China) using the Illumina Novaseq 6000 instrument.

The raw data were processed in Perl and the data quality was checked with FastQC v0.11.2. The mapped genome data were annotated using the GFF3 file provided by Huang et al. [27]. The expression level of transcripts was evaluated by calculating the fragments per kilobase of the exon model per million mapped reads (FPKM) in Perl. software was used to screen differentially expressed genes between different groups. The thresholds for determining DETs were *p* < 0.05 and absolute fold change ≥ 2. Then, the identified DETs were used for functional annotation and pathway enrichment analysis using the Gene Ontology (GO) and Kyoto Encyclopedia of Genes and Genomes (KEGG) databases, respectively. Significantly enriched pathways were determined at *p* < 0.05 and at least two related genes were included.

### 2.6. Immunohistochemical Assays

Mouse brain tissue was fixed with 4% formaldehyde for 24 h, transferred to a 30% sucrose solution until the tissue sank, was frozen and then sectioned into 10-µm-thick slices. The tissue sections were fixed on slides, which were washed three times with PBS solution for 5 min each time, blocked with blocking solution at 25 °C for 90 min, and incubated with primary antibody anti-p-p38, 1:200, CST, 4511; anti-p-NFκBp65, 1:200, CST, 3033T; anti-Iba1, 1:1000, Servicebio, GB12105) at 4 °C overnight. The slides were washed three times with PBS solution for 5 min each time and incubated with the secondary antibody for 1 h away from the light at room temperature. Next, the slides were washed three times with PBS solution for 5 min each time and stained with DAPI. After a final wash with PBS, an anti-quenching agent was added dropwise to mount the slide.

### 2.7. Western Blotting

After deep anesthesia, the mice were sacrificed by decapitation and the cerebral cortex tissue was collected. The cortical tissue was lysed using a high-throughput tissue grinder and lysis mixing buffer (RIPA+PMSF+ protease inhibitors), and the samples were left on crushed ice for 10 min. After centrifugation at 12,000 r/min for 15 min at 4 °C, the supernatant was separated according to the manufacturer’s instructions, and the sample protein concentration was determined by the BCA Protein Assay kit (BOSTER, Wuhan, China). The same amount of protein was separated using electrophoresis on SDSPAGE gel (BOSTER, Wuhan, China) and transferred to a PVDF membrane at constant pressure. The membranes were blocked with 5% skim milk for 120 min at room temperature and washed with TBST. The membranes were cut according to different molecular weight proteins and the corresponding primary antibodies (anti-p38: CST, 8690; anti-p-p38: CST, 4511; anti-MSK: CST, 3489S; anti-p-MSK: CST, 9595S; anti-NFκBp65: CST, 8242; anti-p-NFκBp65: CST, 3033T; anti-GAPDH: BOSTER, A00227-1) and the membranes were placed in antibody diluent using primary antibody diluent (BOSTER, Wuhan, China) diluted at a ratio of 1:1000 and incubated at 4 °C overnight. The next day, the membranes were washed three times with TBST for 5 min each time and incubated with the corresponding secondary antibody (BOSTER, Wuhan, China) for 2 h at room temperature. Finally, all target membrane bands were imaged using a gel imaging system (Bio-Rad, San Francisco, CA, USA) and the gray value of the target band was analyzed using Quantity one software.

### 2.8. Enzyme-Linked Immunosorbent Assay (ELISA)

The concentrations of TNF-α, IL-1β, IL-6, and IL-10 in the cortical tissue of mice were measured using ELISA kits (BOSTER, Wuhan, China) according to the manufacturer’s instructions. The concentrations of COX2 and iNOS in the cortical tissue were measured using ELISA kits (Elabscience Biotechnology, China) according to the manufacturer’s instructions.

### 2.9. Superoxide Dismutase Activity

Superoxide dismutase (SOD) activity in the cortical brain tissue was measured using an SOD assay kit (Beyotime, Shanghai, China) per the manufacturer’s instructions.

### 2.10. Data Analysis

All data are presented as the mean ± standard error of the mean (SEM). The data were analyzed in SPSS (version 26.0, IBM Corp., Armonk, NY, USA). All data were tested for normal distribution and homogeneity of variance. Between-group differences were analyzed with a Student’s *t*-test. Multi-group comparisons were performed with one-way analysis of variance (ANOVA). The Least Significant Difference (LSD) was used for multiple comparisons of data that met the homogeneity of variance in the one-way analysis of variance. Statistical significance was indicated at *p* < 0.05. The data were visualized with GraphPad Prism 8.3.0 (GraphPad Software, Inc., San Diego, CA, USA).

## 3. Results

### 3.1. Effect of SD on Emotional Behavior in Mice

**Figure 2:**
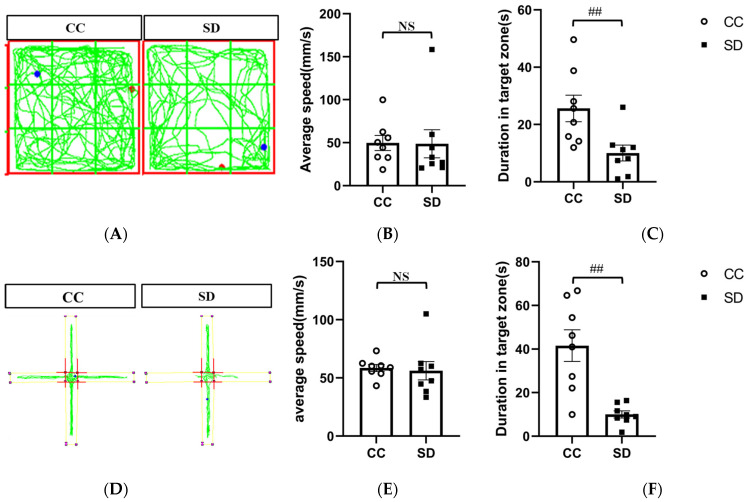
Effects of sleep deprivation on emotional behavior in mice. (**A**) Representative track plot of mice in the open-field test. (**B**,**C**) Results of the open-field test. CC, *n* = 8; SD, *n* = 8. (**D**) Representative track plot of mice in the elevated plus maze experiment. (**E**,**F**) Results of the elevated plus maze experiment. CC, *n* = 8; SD, *n* = 8. Data shown are mean ± SEM. Data were analyzed by one-way ANOVA (**B**,**C**,**E**,**F**).; ## *p* < 0.01 vs. CC; NS, no significance.

We evaluated the effect of SD on the emotional behavior of mice using the open-field and elevated plus maze experiments. In the open-field experiment, mice in the SD group spent significantly less time in the open central area than mice in the CC group (*p* < 0.01). Similarly, in the elevated plus maze experiment, mice in the SD group spent significantly less time in the open arm than mice in the CC group (*p* < 0.01). The movement speed of the mice did not change significantly, indicating that this SD model triggers anxiety-like emotional behavior in the mice without affecting their mobility (Figure 2). Results for non-target zones are presented in the Supplementary Material (Supplementary Figure S1).

### 3.2. Effect of SD on the Transcriptome of the Prefrontal Cortex

**Figure 3:**
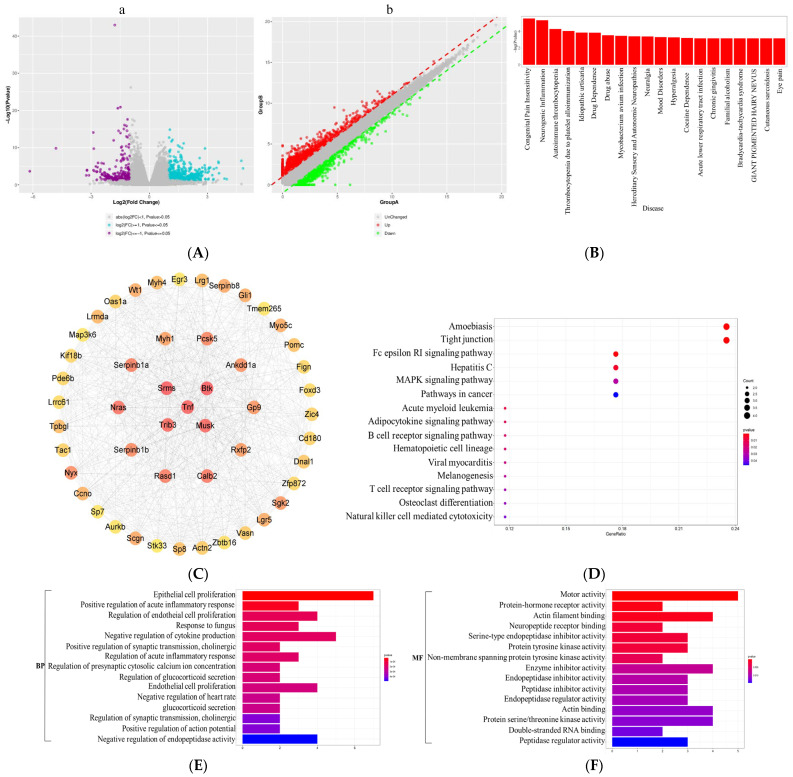
Effects of sleep deprivation on the transcriptome of the prefrontal cortex in mice. (**A**) Volcano map (**a**) and scatter map (**b**) showed differentially expressed genes (DEGs) in the prefrontal cortex of SD vs. CC mice (*n* = 4). (**B**) DEGs disease annotation analysis in the prefrontal cortex of SD vs. CC mice. (**C**) Top 50 DEGs in the prefrontal cortex of SD vs. CC mice. (**D**) KEGG-based enrichment analysis of the top 50 DEGs in the prefrontal cortex of SD vs. CC mice. (**E**) GO-based Biological Process analysis of the top 50 DEGs. (**F**) GO-based Molecular Function analysis of the top 50 DEGs.

To further investigate the molecular mechanisms of SD-induced anxiety-like emotional behavior in mice, we performed a transcriptome sequencing analysis of mouse prefrontal cortex tissue. SD led to the upregulation of 297 genes and downregulation of 578 genes (Figure 3A). The results of enrichment analysis showed that these differentially expressed genes (DEGs) were strongly associated with neurogenic inflammation, substance abuse, pathological neuralgia, and mood disorders (Figure 3B). The top 50 DEGs with respect to degree of interaction were extracted using cytoHubba to generate a protein–protein interaction (PPI) network (Figure 3C). KEGG-based functional enrichment analysis of the top 50 DEGs revealed that the DEGs were closely associated with amoebiasis, hepatitis C, and the MAPK pathway. In particular, the Tnf/Nras/Map3k6 genes were significantly enriched in the MAPK pathway. GO-based Biological Process (GOBP) analysis showed that the DEGs were closely associated with epithelial cell proliferation, positive regulation of acute inflammatory responses, and negative regulation of cytokine production. A GO-based Molecular Function (GOMF) analysis indicated that the DEGs were closely associated with motor activity, protein–hormone receptor activity, and protein tyrosine kinase activity. Transcriptome sequencing of the prefrontal cortex of the mouse brain indicated that, compared with CC mice, SD mice showed differential gene expression closely associated with the MAPK pathway (Figure 3).

### 3.3. Effect of SD on the Activation of the p38/MSK1/NFκB Pathway

**Figure 4:**
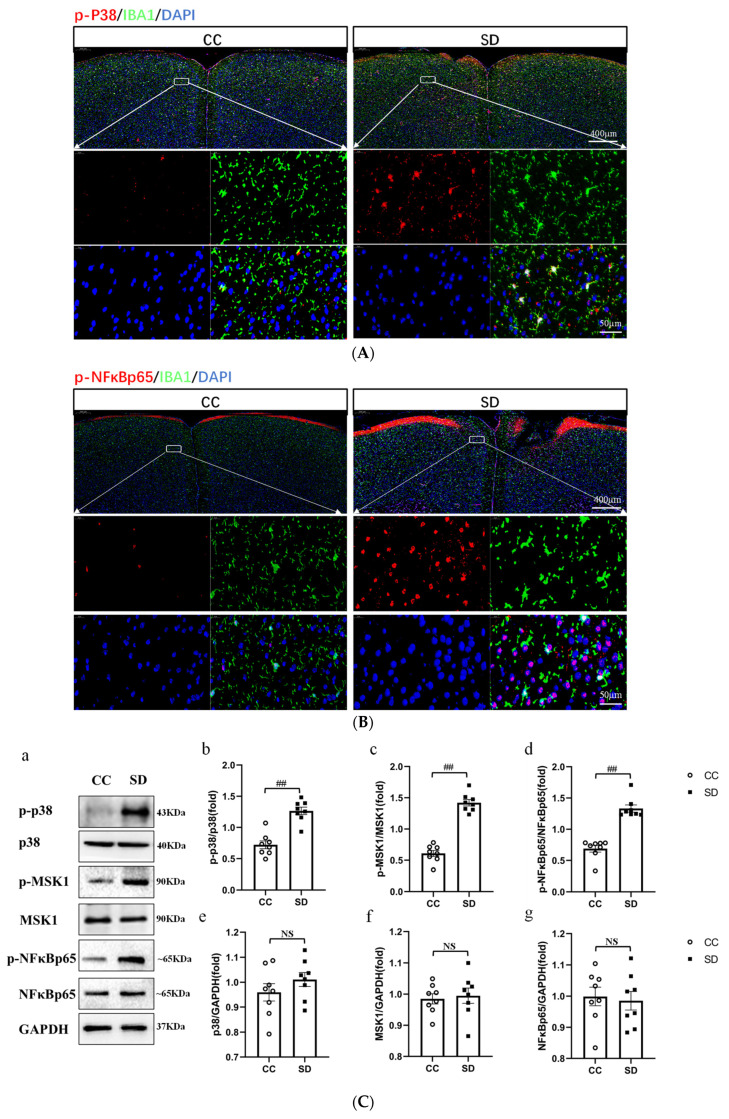
Effects of sleep deprivation on the activation of the p38/MSK1/ NF-κB pathway. (**A**) Immunofluorescence in cells co-labeled with p-p38 (red) and IBA1 (green). (**B**) Immunofluorescence in cells co-labeled with p-NFκBp65 (red) and IBA1 (green). The white dashed arrow points to a higher magnification of the area in the white box. Scale bars: low magnification, 400 µm; high magnification, 50 µm. (**C**) (**a**) Western blot analysis of proteins in the p38/MSK1/NFκB pathway in the prefrontal cortex of mice; (**b**–**d**) Expression levels of phosphorylated proteins after standardization with respect to total protein expression; (**e**,**f**) Total protein expression after standardization with respect to GAPDH expression. CC, *n* = 8; SD, *n* = 8. Data shown are mean ± SEM. Data were analyzed by one-way ANOVA (**b**–**d**,**f**,**g**). ## *p* < 0.01 vs. CC; NS, no significance.

To investigate whether the p38/MSK1/NFκB pathway is involved in the molecular mechanism of SD leading to anxiety-like mood changes in mice, we analyzed the prefrontal cortex tissues of mice with immunofluorescence staining for p-p38, p-NFκBp65, and IBA1 (a marker of microglia activation). The results showed that the prefrontal cortex tissues of SD mice had more numerous activated microglia than those of CC mice. In addition, the expression of p-p38 and p-NFκBp65 was higher in SD mice, and some of the fluorescently labeled cells were co-localized with activated microglia (Figure 4A,B). Western blotting experiments were performed to confirm whether SD activated the p38/MSK1/NFκB pathway and the results indicated no differences in total expression levels of the proteins (p38, MSK, and NFκBp65) in the prefrontal cortex tissues of SD vs. CC mice. However, the expression levels of their respective phosphorylated (activated) forms—p-p38, p-MSK, and p-NFκBp65—were significantly higher (*p* < 0.01) in SD mice, indicating that the SD model promotes the activation of the p38/MSK1/NFκB pathway (Figure 4C).

### 3.4. SB203580 Can Effectively Inhibit the Activation of p38/MSK1/NFκB Pathway Induced by SD

**Figure 5:**
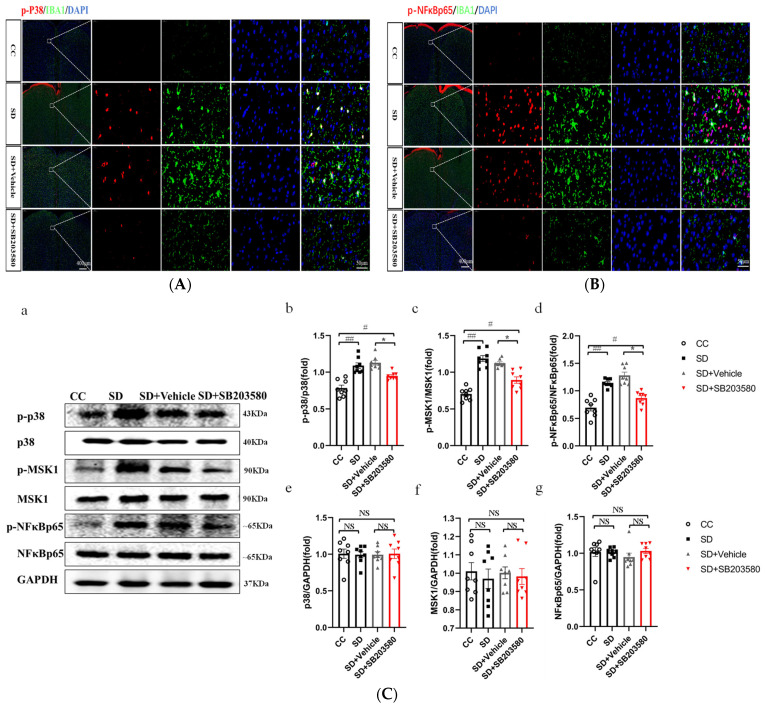
SB203580 inhibits the activation of the p38/MSK1/NF-κB pathway. (**A**) Immunofluorescence in cells co-labeled with p-p38 (red) and IBA1 (green). (**B**) Immunofluorescence in cells co-labeled with p-NFκBp65 (red) and IBA1 (green). The white dashed arrow points to a higher magnification of the area in the white box. Scale bars: low magnification, 400 µm; high magnification, 50 µm. (**C**) (**a**) Western blot analysis of proteins in the p38/MSK1/NFκB pathway in the prefrontal cortex of mice; (**b**–**d**) Expression levels of phosphorylated proteins after standardization with respect to total protein expression; (**e**–**g**) Total protein expression after standardization with respect to GAPDH expression. CC, *n* = 8; SD, *n* = 8. Data shown are mean ± SEM. Data were analyzed by one-way ANOVA (**b**–**d**,**f**,**g**). ## *p* < 0.01 vs. CC; # *p* < 0.05 vs. CC; * *p* <0.05 vs. SD; NS, no significance.

To explore whether the p38/MSK1/NFκB pathway plays an important role in the cortical inflammation and oxidative stress response induced by SD in mice using SB203580, a p38MAPK inhibitor. we first performed immunofluorescence staining for p-p38, p-NFκBp65, and IBA1 in the prefrontal cortex tissues of mice in each group. The prefrontal cortex tissues of SD mice had more numerous activated microglia than those of mice in the CC group. The expression levels of p-p38 and p-NFκBp65 were higher in SD mice, and some of the fluorescently labeled cells co-localized with the activated microglia. The SD + SB203580 group had fewer activated microglia and fewer p-p38- and p-NFκBp65-positive cells than the SD + Vehicle group (Figure 5A,B). Western blotting analysis showed no between-group differences in the total expression levels of the p38 [F = 0.022], MSK [F = 0.122] and NFκBp65 [F = 0.653] proteins in the prefrontal cortex tissues of mice (*p* > 0.05). However, the expression levels of the phosphorylated forms of these proteins (p-p38 [F = 19.059], p-MSK [F = 34.898], and p-NFκBp65 [F = 29.146]) were significantly higher in the SD group than in the control group (*p* < 0.01) and significantly lower in the SD + SB203580 group than in the SD + Vehicle group (*p* < 0.05) (Figure 5C).

### 3.5. SB203580 Ameliorates Oxidative Stress and Inflammatory Reactions in the Prefrontal Cortex of Sleep-Deprived Mice

**Figure 6:**
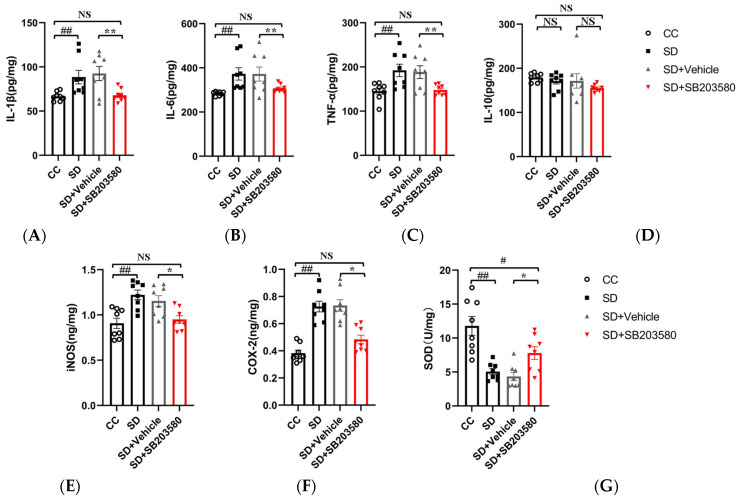
SB203580 alleviates oxidative stress and inflammatory responses in the prefrontal cortex of sleep-deprived mice. (**A**–**F**) ELISA to detect the expression of IL-1, IL-6, TNF-α, IL-10, iNOS, and COX-2 in the prefrontal cortex of mice (*n* = 8). (**G**) SOD activity (*n* = 8). Data shown are mean ± SEM. Data were analyzed by one-way ANOVA (**A**–**G**). ## *p* < 0.01 vs. CC; # *p* < 0.05 vs. CC; ** *p* < 0.01 vs. SD + Vehicle; * *p* < 0.05 vs. SD + Vehicle; NS, no significance.

ELISA was used to detect the secretion of inflammatory markers and the expression of oxidative stress-related factors in the cortical tissues of mice. These data were used to evaluate the effects of SB203580 on oxidative stress and inflammatory responses in the prefrontal cortex of SD mice. The levels of pro-inflammatory factors (IL-1β [F = 5.488], IL-6 [F = 4.462], and TNF-α [F = 5.255]) in the cortical tissues of mice were significantly higher in the SD group than in the CC group (*p* < 0.01) and lower in the SD + SB203580 group than in the SD + Vehicle group (*p* < 0.05). However, there were no statistically significant differences in levels of the anti-inflammatory factor, IL-10 [F = 1.243] (*p* > 0.05). The expression levels of oxidative stress-related factors (iNOS [F = 5.508] and COX-2 [F = 18.643]) were significantly higher in the SD group than in the CC group (*p* < 0.01) and lower in the SD + SB203580 group than in the SD + Vehicle group (*p* < 0.05). Total SOD activity [F = 11.883] in the cerebral cortex tissue was significantly lower in the SD group than in the CC group (*p* < 0.01) and higher in the SD + SB203580 group than in the SD + Vehicle group (*p* < 0.05). Taken together, this suggests that SB203580 alleviates the SD-induced increase in inflammatory responses and oxidative stress in mice (Figure 6).

### 3.6. Effect of Dex on Anxiety-like Behaviors in Sleep-Deprived Mice

**Figure 7:**
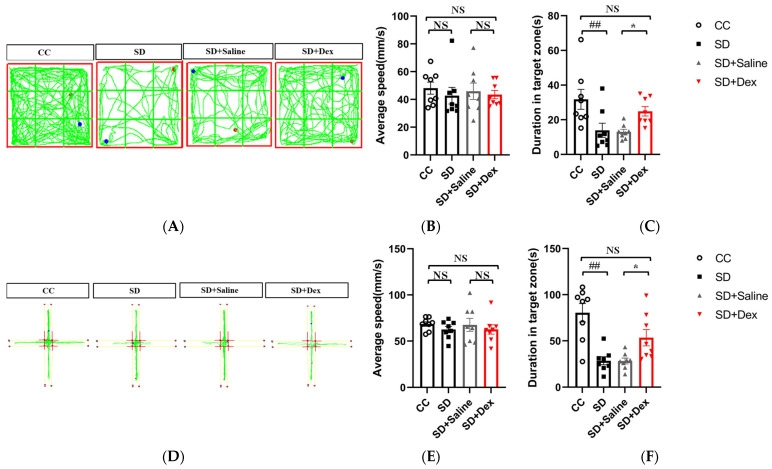
Effects of dexmedetomidine on anxiety-like behaviors in sleep-deprived mice. (**A**) Representative track plot of mice in the open-field test. (**B**,**C**) Results of the open-field test (*n* = 8). (**D**) Representative track plot of mice in the elevated plus maze test. (**E**,**F**) Results of the elevated plus maze test (*n* = 8). Data shown are mean ± SEM. Data were analyzed by one-way ANOVA (**B**,**C**,**E**,**F**). NS, no significance; ## *p* < 0.01 vs. CC; * *p* < 0.05 vs. SD + saline.

Dex improves memory impairment [23] and mood disturbances [22] in sleep-deprived mice by attenuating inflammatory responses. Since Dex has known sedative effects, we determined whether it could improve anxiety-like emotional behaviors in the SD model. The effect of Dex on emotional behavior in sleep-deprived mice was evaluated using the open-field and elevated plus maze experiments. In the open-field experiment [F = 4.880], SD mice spent less time in the central square than CC mice (*p* < 0.01). In contrast, mice in the SD + Dex group spent more time in the central square than mice in the SD + saline group (*p* < 0.05). In the elevated plus maze experiment [F = 10.523], time spent in the open arms was significantly lower in the SD group than in the CC group (*p* < 0.01) but higher in the SD + Dex group than in the SD + saline group (*p* < 0.05). These results indicated that Dex effectively improves anxiety-like behaviors in SD mice. There was no significant change in movement speed between groups, indicating that Dex does not affect the mobility of mice (Figure 7). Results for non-target zones are presented in the Supplementary Material (Supplementary Figure S1).

### 3.7. Dex Acts by Inhibiting the Activation of the p38/MSK1/NFκBp65 Pathway

**Figure 8:**
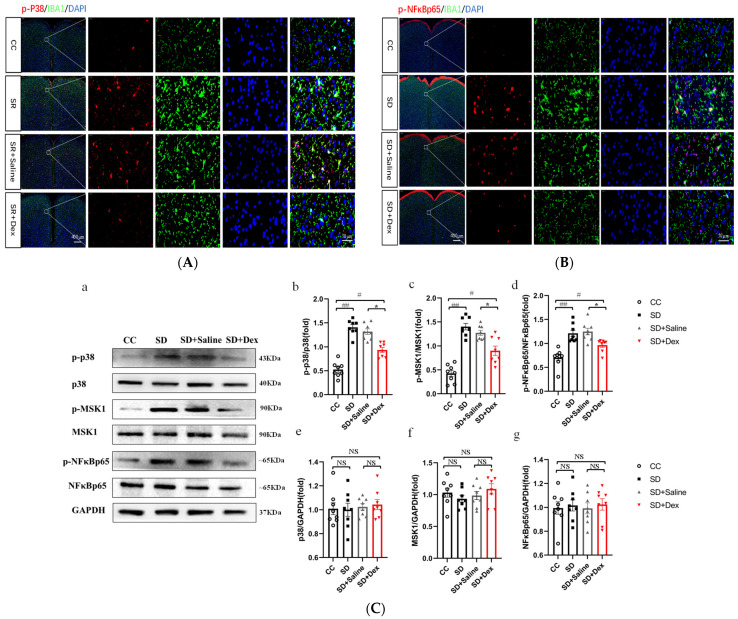
Dexmedetomidine inhibits the activation of the p38/MSK1/NF-κB pathway. (**A**) Immunofluorescence in cells co-labeled with p-p38 (red) and IBA1 (green). (**B**) Immunofluorescence in cells co-labeled with p-NFκBp65 (red) and IBA1 (green). The white dashed arrow points to a higher magnification of the area in the white box. Scale bars: low magnification, 400 µm; high magnification, 50 µm. (**C**) (**a**) Western blot analysis of proteins in the p38/MSK1/NFκB pathway in the prefrontal cortex; (**b**–**d**) Expression levels of phosphorylated proteins after standardization with respect to total protein expression; (**e**,**f**) Total protein expression after standardization with respect to GAPDH expression. CC, *n* = 8; SD, *n* = 8. Data shown are mean ± SEM. Data were analyzed by one-way ANOVA (**b**–**d**,**f**,**g**). ## *p* < 0.01 vs. CC; # *p* < 0.05 vs. CC; * *p* < 0.05 vs. SD + saline; NS, no significance.

To investigate whether Dex acts by inhibiting the activation of the p38/MSK1/NFκB pathway, we first performed immunofluorescence staining for p-p38, p-NFκBp65, and IBA1 in the prefrontal cortex tissues of mice in each group. The prefrontal cortex tissues of SD mice had more numerous activated microglia than those of mice in the CC group. The expression levels of p-p38 and p-NFκBp65 were higher in SD mice, and some of the fluorescently labeled cells co-localized with the activated microglia. The SD + Dex group had fewer activated microglia and fewer p-p38- and p-NFκBp65-positive cells than the SD + saline group (Figure 8A,B). Western blotting analysis showed no between-group differences in the total expression levels of the p38 [F = 0.737], MSK [F = 0.568], and NFκBp65 [F = 0.167] proteins in the prefrontal cortex tissues of mice (*p* > 0.05). However, the expression levels of the phosphorylated forms of these proteins (p-p38 [F = 52.053], p-MSK [F = 36.140], and p-NFκBp65 [F = 10.855]) were significantly higher in the SD group than in the control group (*p* < 0.01) and significantly lower in the SD + Dex group than in the SD + saline group (*p* < 0.05) (Figure 8C).

### 3.8. Dex Ameliorates Oxidative Stress and Inflammatory Reactions in the Prefrontal Cortex of Sleep-Deprived Mice

**Figure 9:**
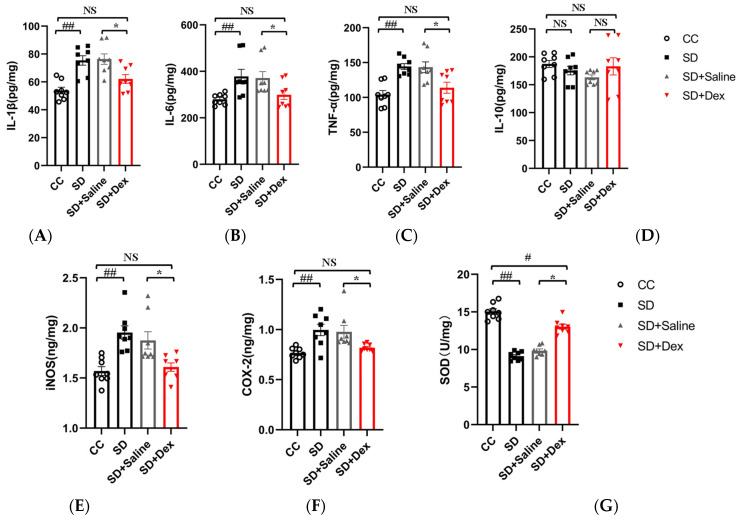
Dexmedetomidine alleviates oxidative stress and inflammatory responses in the prefrontal cortex of sleep-deprived mice. (**A**–**F**) ELISA to detect the expression of IL-1, IL-6, TNF-α, IL-10, iNOS, and COX-2 in the prefrontal cortex of mice (*n* = 8). (**G**) SOD activity (*n* = 8). Data shown are mean ± SEM. Data were analyzed by one-way ANOVA (**A**–**G**). # *p* < 0.05 vs. CC; ## *p* < 0.01 vs. CC; * *p* < 0.05 vs. SD + saline; NS, no significance.

ELISA was used to detect the secretion of inflammatory markers and expression of oxidative stress-related factors in the cortical tissues of mice. These data were used to evaluate the effects of Dex on oxidative stress and inflammatory responses in the prefrontal cortex of SD mice. The levels of pro-inflammatory factors (IL-1β [F = 9.289], IL-6 [F = 4.523], and TNF-α [F = 8.018]) in the cortical tissues of mice were significantly higher in the SD group than in the CC group (*p* < 0.01) and lower in the SD + Dex group than in the SD + saline group (*p* < 0.05). However, there were no statistically significant differences in levels of the anti-inflammatory factor, IL-10 [F = 1.121] (*p* > 0.05). The expression levels of oxidative stress-related factors (iNOS [F = 9.649] and COX-2 [F = 6.804]) were significantly higher in the SD group than in the CC group (*p* < 0.01) and lower in the SD + Dex group than in the SD + saline group (*p* < 0.05), with the difference in iNOS levels being highly significant (*p* < 0.05). Total SOD activity [F = 52.316] in the cerebral cortex tissue was significantly lower in the SD group than in the CC group (*p* < 0.01) and higher in the SD + Dex group than in the SD + Saline group (*p* < 0.05). Taken together, this suggests that Dex alleviates the SD-induced increase in inflammatory responses and oxidative stress in mice (Figure 9).

## 4. Discussion

REM SD protocols (or paradoxical SD protocols) are the most frequently used methods of SD [28]. The modified multiple platform (MMP) method [11,29,30] is a common form of REM SD that overcomes the shortcomings of unstable experimental animal populations. However, the animals are still affected by stress from movement restriction, resulting in environmental confounding factors. Researchers have recently developed a novel SD protocol for mice, consisting of a cylinder and built-in bar. The mice are allowed to move freely in the cylinder with ad libitum access to food and water. To induce SD, the bar inside the cylinder is rotated continuously at a constant speed [31]. This type of SD neither isolates the mice nor restricts their mobility. The results showed that this sleep deprivation model induced anxiety-like behaviors in mice (Figure 2), which was consistent with previous findings [10,32].

Evidence from human studies suggests that the prefrontal cortex (PFC) plays an important role in sleep [33] and anxiety [34]. The impairment of PFC activity after sleep deprivation is closely related to the anxiety induced by sleep deprivation and can predict the degree of anxiety amplification [35]. Moreover, it has been demonstrated that the activation of microglia in the PFC underlies anxiety-like behaviors in sleep-deprived mice [10]. Therefore, to further investigate the molecular mechanisms underlying SD-induced anxiety-like emotional behaviors in mice, we performed transcriptome sequencing analysis using the prefrontal cortex tissue of mice. Several DEGs with strong interactions were significantly enriched in the MAPK pathway and were closely associated with positive regulation of acute inflammatory response and negative regulation of cytokine production (Figure 3). Further validation of the sequencing results revealed that SD leads to the activation of the p38/MSK1/NFκB pathway (Figure 4). The MAPK family includes ERK1/2, JNK, p38, and ERK5. Of these, JNK and p38 can be activated by intracellular and extracellular stress (including changes in environmental factors such as UV, heat, and hyperosmotic stress) and inflammatory cytokines [36]. As mentioned above, the p38 MAPK pathway appears to be a key link between pathological microglia activation and deleterious inflammation in CNS disease [37]. MSK1 and 2 are two related kinases that are activated downstream of p38 and Erk1/2 [38], and MSK1 can prolong the activation of NFκB [39]. The p38 and ERK-MAPK signaling pathways are involved in the SD-induced activation of the NLRP3/pyroptosis axis [25]. Insufficient sleep also induces other pro-inflammatory factors such as IL-1β and TNF-α, which can activate p38 MAPK and are involved in the inhibition of neural precursor cells [24]. Our results confirmed that SD leads to the activation and phosphorylation of p38 and MSK1, further leading to NFκB activation, oxidative stress, and inflammatory responses. We further confirmed the important role of the p38/MSK1/NFκB pathway in SD-induced neuroinflammatory response using the p38 inhibitor SB203580 (Figure 5 and Figure 6).

Recent studies have shown that Dex can alleviate lung injury in septic mice by regulating the p38 MAPK signaling pathway [40]. Dex also alleviates lipopolysaccharide-induced apoptosis in hippocampal neurons by reducing the level of inflammatory factors (such as IL-1β, TNF-α, and IL-6) through phosphorylation of the p38 MAPK pathway [41]. These lines of evidence demonstrate that Dex plays a neuroprotective role by modulating the p38 MAPK pathway. Dex attenuates the SD-induced exacerbation of postoperative immunosuppression [29] and improves memory impairment [23] and depression in sleep-deprived mice by attenuating the inflammatory response [22]. Our results showed that Dex ameliorated anxiety in SD mice by inhibiting the activation of the p38/MSK1/NFκB pathway in microglia (Figure 7 and Figure 8).

Long-term SD (e.g., due to short sleep duration or sleep disorders) can lead to chronic systemic low-grade inflammation. It is also associated with several diseases with inflammatory aspects such as diabetes, atherosclerosis, and neurodegeneration [7]. Astrocytic phagocytosis of synaptic elements (mainly the presynaptic components of large synapses) is increased after both acute and chronic SD (compared with sleep and wakefulness). Moreover, low levels of sustained microglia activation can lead to abnormal responses to secondary injury. Thus, chronic SD initiated by the microglia may render the brain vulnerable to further injury [9]. In the present study, we found that microglia activation was caused by SD (Figure 4) and inhibited by Dex (Figure 8). The cytokines IL-1β and TNF-α are both involved in the regulation of sleep homeostasis [42,43]. Animal studies have shown that most pro-inflammatory cytokines promote NREM sleep, whereas anti-inflammatory cytokines reduce NREM sleep; in addition, the inhibition of the activity of certain inflammatory cytokines improves sleep quantity and quality [7]. Recent studies have shown that MMP-induced sleep deprivation for 72 h increases the levels of pro-inflammatory cytokines (IL-1β, IL-6, and TNF-α) and reduces the levels of anti-inflammatory cytokines (IL-4 and IL-10) in rat hippocampal tissue [44]. MMP-induced sleep deprivation for 20 h per day for 7 days resulted in increased levels of TNF-a, IL-1β, and IL-6 in mice. Similar results were obtained in the present study, where SD led to increased secretion of the pro-inflammatory factors IL-1β, IL-6, and TNF-α (but had no effect on IL-10 levels; Figure 9). Dex has been shown to ameliorate SD-induced decreases in short-term memory and spatial learning in rats by inhibiting the SD-induced production of inflammatory mediators (TNF-α and IL-6) [23]. Dex also inhibits the production of TNF-α, IL-1β, and IL-18, the phosphorylation of ERK1/2 and P38, and the activation of caspase-1, and reduces pyroptosis [45]. These results are consistent with the findings of this study, where we show that Dex administration reduced the SD-induced secretion of pro-inflammatory factors.

One of the functions of sleep is to promote antioxidant mechanisms. This may be an adaptive response to sleep deficiency/deprivation, which can induce oxidative stress [28]. Recent findings suggest that the duration of sleep fragmentation is a major factor in the development of anxiety-related behaviors and that these effects are mediated through oxidative stress in the brain [46]. Chronically sleep-deprived rats exhibited reduced SOD activity in the hippocampus and brainstem [47]. In contrast, acutely sleep-deprived rats (6 h) exhibited reduced glutathione (GSH) levels in the cortex, brainstem, and forebrain and enhanced glutathione peroxidase (GPx) activity in the hippocampus and cerebellum after mild treatment [48]. These findings are consistent with the results of the present study (Figure 9G). However, studies on other SD animal models have not reported any changes in oxidative stress markers or antioxidant capacity in the peripheral blood or brain regions after SD [49,50]. These inconsistencies may be associated with the different methods and durations of sleep deprivation and the mouse strains used in the SD experiments.

The present study has some limitations. On one hand, we found that the effect of SB203580 on anxiety behavior was not statistically significant (Supplementary Figure S2). The possible reason was that the duration of inhibitor use was not enough. We refer to previous literature [26] using SB203580 for up to 14 days. Another possible reason is that there are other important signaling pathways in the anxiety behavior caused by SD, which is also the direction of our future research. On the other hand, only male mice were used in this experiment and we did not test for any between-sex differences in the ameliorating effects of Dex on emotional behavior in sleep-deprived mice. However, many drugs are known to have sex-dependent effects [51], and the associations between stress, sleep deprivation, and inflammation appear to be stronger in females than in males. Although sex is typically considered a confounder, future studies should investigate differences between the sexes in a more systematic manner [52].

## 5. Conclusions

**Figure 10:**
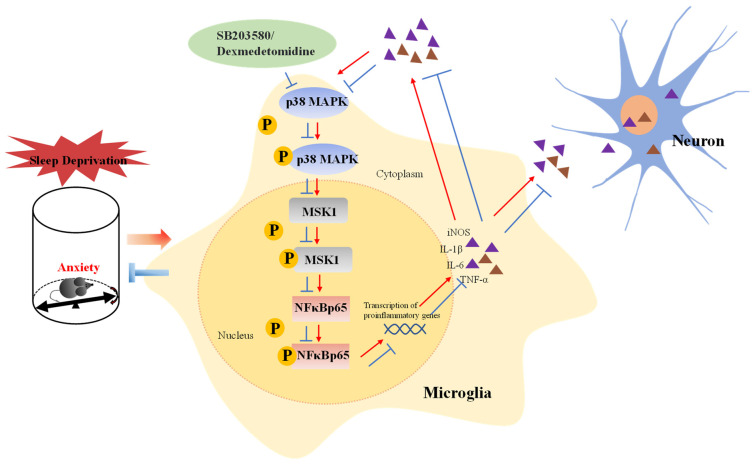
Mechanism of Dex in relieving anxiety caused by sleep deprivation. In this study, a mouse model of sleep deprivation was established using an interference rod device, which induced anxiety in mice. The mechanism may be related to the activation of the P38/MSK1/NFκB signaling pathway in the damaged cortex of SD mice. At the same time, microglia are significantly activated and central inflammation ensues (the schematic is shown with red arrows). Dexmedetomidine can inhibit the inflammatory response, inhibit the activation of the P38/MSK1/NFκB signaling pathway, and finally alleviate the anxiety-like behavior of SD mice (the schematic is shown with blue lines).

In summary (Figure 10), the present study demonstrates that a novel sleep deprivation instrument can trigger anxiety-like behaviors in mice. Transcriptome sequencing and other experiments confirmed that SD leads to the activation of the p38/MSK1/NFκB pathway. In addition, Dex inhibits the activation of the p38/MSK1/NFκB pathway, thus attenuating SD-induced inflammatory responses and oxidative stress in the cerebral cortex of mice. These insights provide a theoretical basis for using Dex in the treatment of patients with insomnia and insomnia-induced mood disorders. Moreover, we provide potential research targets for further investigation of the molecular mechanisms underlying the Dex-induced improvement of SD-induced anxiety-like emotional behaviors.

## Data Availability

The analysis data used to support the findings of this study are available from the corresponding author upon request.

## Supplementary Materials

The following supporting information can be downloaded at: https://www.mdpi.com/article/10.3390/brainsci13071058/s1, Figure S1: Information about the speeds and times spent in the non-target zones; Figure S2: (A,B) Results of the open-field test. (C,D) Results of the elevated plus maze test.
