# Supplementary material for: Dexmedetomidine Improves Anxiety-like Behaviors in Sleep-Deprived Mice by Inhibiting the p38/MSK1/NFκB Pathway and Reducing Inflammation and Oxidative Stress
Source: Brain Sci. 2023 Jul 11;13(7):1058. doi: 10.3390/brainsci13071058 (PMC10377202; doi:10.3390/brainsci13071058)
Supplement: Supplementary file 1 [file brainsci-13-01058-s001.zip › Supplementary materials.pdf]

Supplementary materials

1. Behavioral data for the non-target zones

In the open-field experiment, mice in the SD group spent more time in the non-target zones than mice in the CC group ($p < 0.05$) (Figure S1 B). Similarly, in the elevated plus maze experiment, mice in the SD group spent significantly more time in t the non-target zones than mice in the CC group ($p < 0.01$) (Figure S1 D). The average speed in the non-target zones did not change significantly (Figure S1 A, C). After adding Dexmedetomidine (Dex), in the open-field experiment, SD mice spent more time in the non-target zones than CC mice ($p < 0.05$). In contrast, mice in the SD + Dex group spent less time in the non-target zones than mice in the SD + saline group ($p < 0.05$) (Figure S1 F). After adding Dex, in the elevated plus maze experiment, time spent in the non-target zones was significantly higher in the SD group than in the CC group ($p < 0.01$) but lower in the SD + Dex group than in the SD + saline group ($p < 0.05$) (Figure S1 H). The average speed in the non-target zones did not change significantly (Figure S1 E, G).

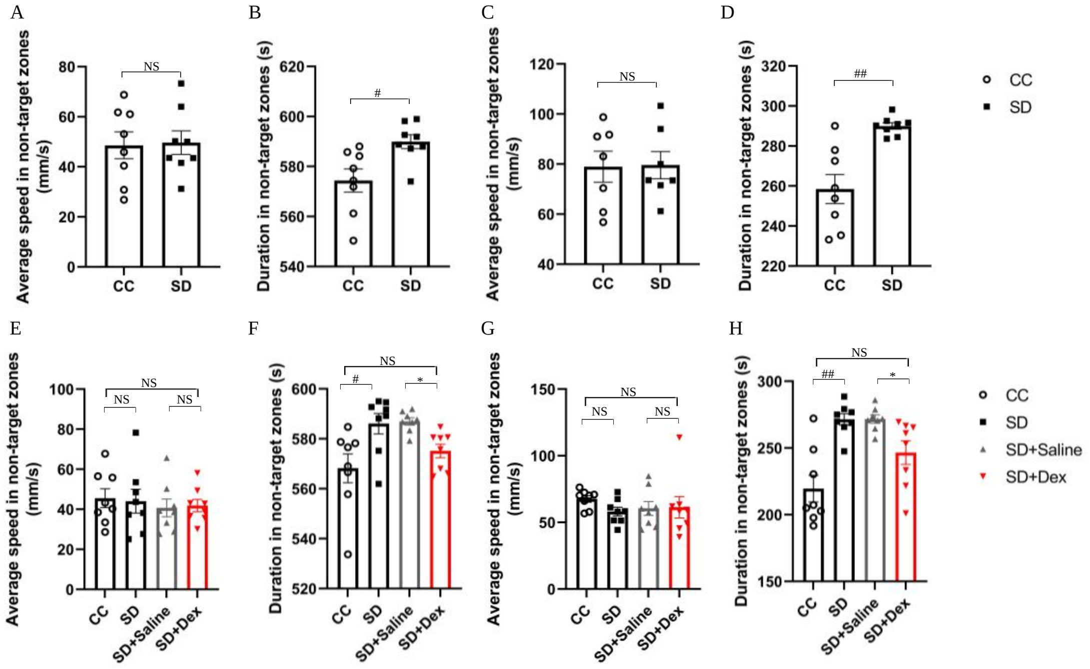

Figure S1. Information about the speeds and times spent in the non-target zones. (A,E) Average speed in the non-target zones of the open field experiment; (B,F) Duration in the non-target zones of the open field experiment; (C,G) Average speed in the non-target zones of the elevated plus maze experiment; (D,H) Duration in the non-target zones of the elevated plus maze experiment. Data shown are mean ± SEM. $^{\#\#}$ $p < 0.01$ vs. CC; $^{\#}$ $p < 0.05$ vs. CC; * $p < 0.05$ vs. SD + saline; NS, no significance.

2. Effect of SB203580 on anxiety-like behaviors in sleep-deprived mice

In the open-field experiment [F=3.891], SD mice spent less time in the central square than CC mice ($p < 0.05$). There was no significant difference in the time spent in the central square between the SD + SB203580 group and the SD + Vehicle group

mice (P>0.05). In the elevated plus maze experiment [F=3.225], time spent in the open arms was significantly lower in the SD group than in the CC group ($p < 0.05$). There was no significant difference in the time spent in the open arms between the SD + SB203580 group and the SD + Vehicle group mice (P>0.05).

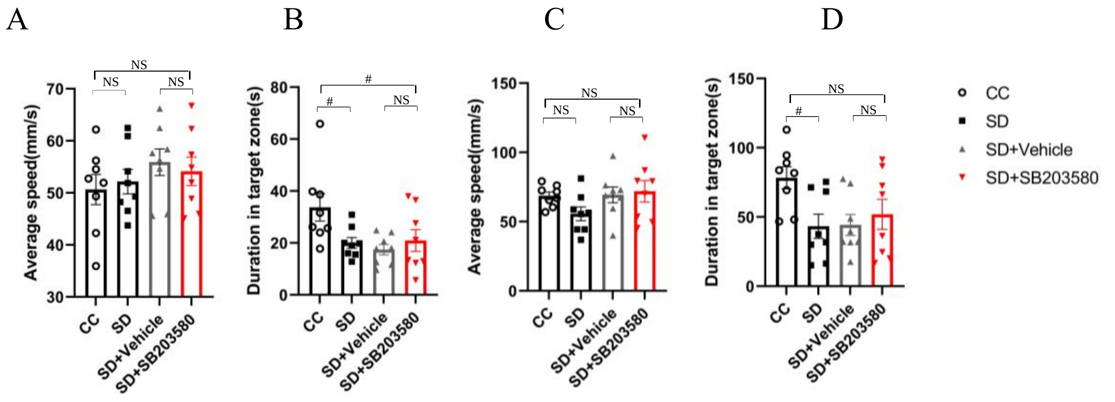

Figure S2. (A,B) Results of the open-field test. (C,D) Results of the elevated plus maze test. # $p < 0.05$ vs. CC; NS, no significance.
